# Relation between Motor and Cognitive Skills in Italian Basketball Players Aged between 7 and 10 Years Old

**DOI:** 10.3390/sports6030080

**Published:** 2018-08-14

**Authors:** Francesca Policastro, Agostino Accardo, Roberto Marcovich, Giovanna Pelamatti, Stefania Zoia

**Affiliations:** 1 Life Science Department, University of Trieste, 34100 Trieste, Italy; pelamatti@units.it (G.P.); stefania.zoia@asuits.sanita.fvg.it (S.Z.); 2 Architecture and Engineering Department, University of Trieste, 34100 Trieste, Italy; accardo@units.it; 3 Medical Science Department, University of Trieste, 34100 Trieste, Italy; marcovic@units.it

**Keywords:** basketball, Movement Assessment Battery for Children-2, attention, visuo-spatial working memory, motor manual sequences

## Abstract

There is evidence supporting a correlation between motor, attention and working memory in children. This present study focuses on children aged between 7 and 10 years, who have been playing basketball in the last two years. The aim of this study is to verify the correlation between cognitive and motor abilities and to understand the importance of this correlation in basketball practice. A total of 75 children who were 7.2–10.99 years old were assessed in terms of their attention, motor manual sequences and visuo-spatial working memory. A regression analysis was provided. In this sample, the motor abilities of children were found to be correlated with attention (denomination task, R^2^ = 0.07), visuo-spatial working memory (R^2^ = 0.06) and motor manual sequencing (aiming and catching task, R^2^ = 0.05; and manual dexterity task, R^2^ = 0.10). These correlations justify the suggestion to introduce deeper cognitive involvement during basketball training. The development of executive functions could have an important impact on basketball practice and the introduction of attention and memory tasks could help coaches to obtain optimal improvement in performance during the training sessions.

## 1. Introduction

Basketball is regarded as one of the most popular sports worldwide. A considerable number of players start practicing basketball as early as 5–6 years of age. In the United States of America (USA), the National Basketball Association and the USA Basketball league created the “Youth Basketball Guidelines” [1], which aimed to promote the physical health of players; to develop age- and stage-appropriate skills; and to foster the development of peer relationships, self-esteem and leadership qualities. These guidelines provide age-appropriate standards that follow the maturation of children. The guidelines focus on the game structure (i.e., game length, timeouts), the tactics (i.e., how to set the defence) and the rules (i.e., how to manage substitutions). In Italy, the basketball association (Federazione Italiana Pallacanestro) involves more than 300,000 coaches, players and young players [2]. In fact, basketball is the second most popular sport after soccer.

Italian basketball coaches for young players complete two-year vocational training before transitioning to practice. During this training period, the focus is largely placed on the physical, social and emotional development of the participating children [3]. Their training needs to include adequate consideration of the aerobic resistance, motor abilities and socio-emotional development of the children [4]. There have been suggestions that training should also engage cognition. For instance, the coaches should incorporate problem-solving games in order to try to foster imaginative processes [5] and develop timing and spacing abilities in their players. In any case, there is a lack of knowledge and specific information that is required for encouraging the cognitive development of these young children during basketball training. Considering the importance of cognitive abilities in motor learning, this lack of knowledge on cognitive skills could have an impact on training. Newell’s Theory of Constraints [6] provides a complete framework for the correlation between cognitive and motor abilities. The type of task, the environment and the capabilities of the individual player influence the motor performance. For instance, the quality of a jump depends on the environmental constraints (i.e., the surface type, the environmental stability), on the task demand (i.e., to jump beyond an object, one/two legs) and on personal characteristics (i.e., strength, cognition, sensitivity). Through this model, Newell demonstrated the reciprocal integration that exists between the dynamic motor and cognitive systems. There is evidence that supports this integration, which was obtained from both healthy [7,8] and clinical samples [9,10]. Focusing on motor control, Coker [11] provided an example of this integration in basketball practice. In fact, motion and cognition can collaborate as an integrated system to provide the motor control of the gestures. Motor control focuses on the neural, physical and behavioural aspects that are necessary to produce the correct movements [12]. In basketball practice, these systems are required to refine and re-learn motor-skills, such as intercepting a ball at the correct time or improving the landing biomechanics to prevent injuries. Prerequisite abilities, such as control precision, multi-limb coordination, rate control, aiming and catching, timing control and dynamic flexibilities, are necessary for learning basketball. In this sense, cognitive and motor systems can be integrated to guarantee the best possible motor performance. Evidence has demonstrated that physical activity has an important impact on the development of the executive functions in children [13,14].

In this study, we hypothesised the opposite as we suggested that the introduction of a cognitive aspect would have an impact on basketball practice of children between 7 and 11 years old. In fact, this study focuses on the importance of a keen consideration of the cognitive aspects during basketball learning and practice. To introduce this type of approach, it is essential to analyse the relations between motor and cognitive skills of this particular sample. The preliminary aim of this study is to verify how the young basketball players normally develop motor and cognitive abilities, especially considering attention and memory functions. The purpose is not to compare basketball players to other athletes but to assess them with respect to the general Italian population. Furthermore, we wanted to understand if and how attention and memory abilities could be stimulated during basketball practice. Considering actual basketball practice, the main aim of this study is to propose the involvement of some specific executive functions tasks during basketball practice. Using the following results, we will be able to acquire a deeper understanding of the development of these young basketball players in order to provide some suggestions to coaches about the relevance of cognitive development in basketball practice.

## 2. Materials and Methods

### 2.1. Procedure

First, the project was sent to and accepted by the Ethic Committee of the University of Trieste. Following ethical approval, four basketball clubs in the region of Trieste (Italy) were invited to participate in this project. The data were collected and shared with the clubs to assess the development of the young players. A total of 116 parents gave their written informed consent to let their children participate in this study. These parents also filled an anamnestic questionnaire about the health status and the development of the child. The participants were assessed during training in an ecological situation but in a quiet and reserved part of the gym. Testing was conducted in two 30–45 min sessions for each child. For each subject, the sessions were both completed in two months, which were always conducted by the same operator. This study started on January 2016 and ended on February 2017. Testing was randomised in order to eliminate order-related biases. The participants showed interest in the project and were motivated.

### 2.2. Participants

A total of 116 participants (79 boys and 37 girls) were recruited. The mean age was 9.23 years (SD = 1.07). The participation during this study depended on the participation of the children in basketball training. For this reason, the final sample included only 75 children (53 boys and 22 girls) who were aged between 7.23 and 10.99 years (mean = 9.36, SD = 0.98).

The inclusion criteria were being children that are aged 7–10.99 years old and playing basketball for at least the last two years. Furthermore, the participants must have practiced basketball two or three times per week at a competitive level. The basketball activity is not linked to the school program and there were no academic penalties if the children did not participate.

Using the parents’ questionnaire, one participant with Attention Deficit Hyperactivity Disorder (ADHD) was excluded from the study.

### 2.3. Measures

To assess motor and cognitive skills, the following neuropsychological tests were used. Each test provides specific instructions to be administered in the correct manner, which allowed us to create trials with different controls.

In order to provide a complete assessment of cognitive and motor skills, the proposed tasks are at different difficulty levels, which are useful in characterising healthy and sporty children.

- Movement Assessment Battery for Children—2 (MABC-2) [15].

The MABC-2 is a standardised test that is used to assess the motor skills of children with movement difficulties in the following domains: Manual dexterity (MD), aiming and catching (AC) and balance (Bal). Cluster and total standard scores for Italian children are provided [16], with higher scores demonstrating better performance. A total test score at or below the 5th percentile indicates significant movement difficulty, while a score between the 5th and 15th percentile indicates that a child is “at risk”.

In these tests, the items and the scores were compared to the normative data of the uploaded version of the Italian MABC-2.

- Attention, Inhibition and Switching Assessment from the Neuropsychological Assessment—2 (NEPSY-II) [17].

This test is included in the “Attention and Executive Functioning” domain. It requires each participant to look at a series of black and white shapes or arrows and name the shape, the direction or an alternate response depending on the colour of the shape or arrow. The subtests of denomination, inhibition and switching provide information about the accuracy of the sample in terms of timing and errors made when completing the tests. A comparison between the plots that show the relationship between time and errors is not possible as many scores were 0.

In these tests, the items and the scores were compared to the normative data of the uploaded version of the Italian NEPSY-II.

- Manual Motor Sequences Assessment (MMSA) from NEPSY-II

This test is included in the “Sensorimotor” domain. “Manual Motor Sequences” requires each participant to imitate and repeat a series of hand movements performed by the examiner. This counts the number of the manual sequences that participants can replicate.

In this test, the items and the scores were compared to the normative data of the uploaded version of the Italian NEPSY-II.

- Corsi’s Test—Sequential Spatial Task [18].

This test assesses visuo-spatial short-term working memory. Each participant should imitate the examiner who taps a sequence of up to nine identical spatially separated blocks. Each participant should also be able to repeat this tapping sequence backwards.

### 2.4. Statistical Analysis

We conducted statistical analysis using Matrix Laboratory (MatLab). First, the sample was compared to the normative data given by the tests in a qualitative and in a quantitative way whenever possible. For the quantitative assessment, we used the unpaired *t*-test (*p*-value < 0.05) to compare the observed data to the full data set of normative values. For MABC-2 and its subtests, the scores are given in percentiles [15]. Thus, there were no comparisons made using *p*-values but qualitative considerations were undertaken, which were related to the incidence of motor deficits. For the three attention tasks, qualitative suggestions are provided due to the structure of the given standardised data, which consider the accuracy in terms of time and error relations [17]. For MMSA and for the Corsi’s Test, it was possible to obtain a quantitative comparison [17,18]. 

Second, the scores obtained by the participants were used to provide a regression analysis of the correlations between MABC-2 and attention, visuo-spatial working memory (Corsi’s Test) and motor manual sequencing (MMSA).

All the chosen tests are internationally validated and used. Furthermore, they have also been adapted and standardised for the Italian population. The differences found between the countries highlight the importance in considering the cultural variables. For this reason, the standard population given by the tests allowed comparison with the subjects of the study. Comparing the sample to a standard expected normative Italian population could partially reduce the limitations of not having a control group.

## 3. Results

### 3.1. Data Analysis and Description

Table 1 shows the means, the standard deviations, the ranges and the expected values for the considered variables. All the scores are age-standardised.

The sample of children in this present study had a mean rank in the 73rd percentile in the MABC-2 test. Two children scored lower than the critical score. Both children had a very low score in the MD cluster (1st percentile). MD is also the cluster with the lower mean-score.

In NEPSY-II attention test, it is important to highlight that the majority of the observed sample had higher ranks in each subtest compared to the standard population (59% in denomination, 60% in inhibition and 64% in switching).

In NEPSY-II MMSA test, the sample demonstrates statistically significant higher scores compared to the standard population provided by the test, with a *p*-value of 0.017 (standard population: mean-score = 10, SD = 3).

In Corsi’s test, the mean-score of the sample was 4.4 (SD = 1.15), with this test having a normal range of 2–7 points. The standardised scores depend on the age of the child and change for every year. A brief stratification of ages and the unpaired *t*-test scores (see Table 2) demonstrated that there were no significant differences between the sample and the normal population. Nine children scored lower than the critical score for their age.

### 3.2. Regression Analysis

Table 3 contains all pairs of variables that had a significant correlation (*p*-value < 0.05). The other correlations were not reported because they do not have an impact on the aim of this study. 

First, Table 3 summarises the regression results for the MMSA by showing just the statistically significant correlations. It can be observed that MMSA is moderately correlated with the total scores of MABC-2, MD and AC.

Second, this table summarises the significant regression results for the attention task performances. The denomination subtest demonstrates a strong correlation with motor skills, while the inhibition subtest is related to MMSA. The switching test is the only subtest of attention that is not related to other variables.

Finally, the table summarises the significant regression results from Corsi’s Test. There still exists a correlation with the motor skills. It is important to emphasise that the two children who scored very low in the MABC-2 test also had low scores on Corsi’s test.

## 4. Discussion

The first aim of this study was to identify the correlations between executive functions and motor abilities in this specific sample. The correlations would support our suggestion that coaches should consider these aspects in training. The description and the analysis of the data demonstrate some positive correlations between motor and cognitive skills in this basketball sample. According to the previous evidence [7,19], the cognitive aspects involved in the development of the motor skills are: Attention [8] (in terms of inhibition and denomination), visuo-spatial working memory [9,10] and sensorimotor ability to imitate motor manual sequences. The results from the present study were consistent with the research of Roebers [20] and Mandlich [21], who demonstrated the involvement of attention in the development of motor skills. We found that performance on the denomination task is correlated with the MABC-2 score (R^2^ = 0.07). For instance, the basketball players frequently utilise their denomination skills when they focus on the number and the position of the player that they are defending. Attention is a necessary executive function for developing correct movement strategies and inhibiting unwanted movement. We found a correlation between the inhibition subtest and the motor manual sequencing (R^2^ = 0.12). Inhibition and motor manual sequencing abilities are involved in the sensorimotor aspect of movement [12], which occurs when the players do not pass the ball to a companion, when an opponent quickly appears or when they stop moving in order to avoid penalties. The correlations of MMSA with AC (R^2^ = 0.05) and MD (R^2^ = 0.10) explain the importance of the motor manual sequencing in implementing a motor action plan. We evaluated these outcomes as being necessary for young basketball players due to the important participation of upper limbs in movement control during basketball practice. For instance, players utilise their ability to imitate motor manual sequences every time they are learning a manual task by imitating the coach, such as during the ball-handling practice. Consistent with the research of Alloway and Temple [9], the present study also demonstrates the significant role of visuo-spatial working memory through the correlation found between the Corsi’s test and the MABC-2 scores (R^2^ = 0.06). Basketball practice is based on visuo-spatial structures, which are used to create and improve complex motor tasks, thus creating “automatic” movements to play. For instance, players use their visuo-spatial working-memory when they learn how to dribble around opponents and when they recall this and other complex automated motor plans during the game. The previous examples highlight how motor and cognitive systems are integrated to guarantee complex controlled motor performance [6]. These capabilities are necessary in the development of basketball practice because they also contribute to the sensorimotor aspect of the movement and permit the development of rapid motor responses during the game [10,21]. The physiological explication of these correlations comes from the shared neural mechanism, especially of cerebellum processes [19,22,23].

The main aim of the study was to consider the introduction of specific cognitive tasks during basketball practice in order to optimise and enrich training potential. Furthermore, children aged between 7 and 11 years old, which formed the sample in this present study, are experiencing important cognitive development as their consciousness of their own cognitive abilities increased in these years. Starting from this developmental period, children are able to identify attention and memory tasks. They can discriminate the meaning and the functioning of the different types of attention and memory [24]. The introduction of cognitive-aware tasks during training could facilitate motor learning itself. Therefore, considering the cognitive dynamic system in basketball learning could provide players with a deeper understanding of their own movements. 

Finally, the results from the present study suggest that coaches should train attention, visuo-spatial working memory and motor manual sequencing ability of their young players. A targeted proposal for young basketball players could help sport-specific learning and could optimise and enrich the players’ abilities.

As mentioned above, we do not want to emphasise that basketball practice is the cause of the correlations found in this study. We want to instead focus on the fact that the presence of these correlations (specific for this basketball sample) could have an impact on basketball learning in young players.

In the future, it could be useful to increase the sample size to confirm the present correlations and to verify whether there is a corresponding increase in their magnitude. It could also be interesting to create and validate a practical training proposal, which would involve training of the executive functions.

The strengths of this study include the practical proposal of cognitive training of children in basketball and the basis of this proposal on the significant statistical correlations found between motor and cognitive skills. The limitations of this present study include the moderate strength of the correlations, which was possibly caused by the small sample size, and the inability to quantitatively analyse all data.

## Figures and Tables

**Table 1:** Means, SDs and ranges of score for the study variables.

Test	Mean	SD	Range
MABC-2 ^^,^*	73	20.9	5–98
MD ^^,^*	57	25.8	1–95
AC ^^,^*	76	18.3	9–99.9
Bal ^^,^*	68	18.0	16–95
Denomination ^^^	11.4	2.8	4–17
Inhibition ^^^	10.4	2.4	6–14
Switching ^^^	10.8	2.7	6–16
MMSA ^^^	10.8	2.3	2–14
Corsi’s Test ^^^	4.4	1.1	2–7

* Score in percentiles, ^^^ Age-standardised score.

**Table 2:** Stratification for ages of Corsi’s Test.

Age	Mean	SD	Expected Mean	*t*-Value	*p*-Value	*n*
10 years	5.00	1.03	4.37	0.85	0.41	20
9 years	3.96	0.95	4.35	0.57	0.57	24
8 years	4.44	1.29	4.22	0.24	0.81	25
7 years	3.83	0.75	4.03	0.35	0.74	6

Note: No significant *p*-values.

**Table 3:** Significant results from regression analysis.

DV	IV	Bs	Bi	SE	Beta	R^2^	*p*-Value
MMSA	MABC-2	3.35	36.85	21.1	0.34	0.12	0.003
MMSA	MD	3.76	14.70	26.1	0.31	0.10	0.006
MMSA	AC	1.97	57.93	20.3	0.22	0.05	0.050
Denomination	MABC-2	2.08	49.58	21.7	0.27	0.07	0.021
Inhibition	MMSA	0.33	7.35	2.2	0.35	0.12	0.002
Corsi’s Test	Inhibition	0.48	8.27	2.4	0.23	0.05	0.046
Corsi’s Test	MABC-2	4.58	53.07	21.9	0.24	0.06	0.042

MMSA = Motor Manual Sequences Assessment; MABC-2 = Movement Assessment Battery for Children-2; MD = Manual Dexterity; AC = Aiming and Catching; DV = dependent variable; IV = independent variable; Bs = Beta slope; Bi = Beta intercept; SE = standard error for Beta; Beta = standardised Beta; and R^2^ = correlation coefficient.
